# Investigating the genetic regulation of the ECF sigma factor σ^S^ in *Staphylococcus aureus*

**DOI:** 10.1186/s12866-014-0280-9

**Published:** 2014-11-30

**Authors:** Whittney N Burda, Halie K Miller, Christina N Krute, Shane L Leighton, Ronan K Carroll, Lindsey N Shaw

**Affiliations:** Department of Cell Biology, Microbiology & Molecular Biology, University of South Florida, Tampa, FL USA; Department of Biological Sciences, Ohio University, Athens, OH USA

**Keywords:** Sigma factor, Regulation, Mutagenesis

## Abstract

**Background:**

We previously identified an ECF sigma factor, σ^S^, that is important in the stress and virulence response of *Staphylococcus aureus*. Transcriptional profiling of *sigS* revealed that it is differentially expressed in many laboratory and clinical isolates, suggesting the existence of regulatory networks that modulates its expression.

**Results:**

To identify regulators of *sigS*, we performed a pull down assay using *S. aureus* lysates and the *sigS* promoter. Through this we identified CymR as a negative effector of *sigS* expression. Electrophoretic mobility shift assays (EMSAs) revealed that CymR directly binds to the *sigS* promoter and negatively effects transcription. To more globally explore genetic regulation of *sigS*, a Tn*551* transposon screen was performed, and identified insertions in genes that are involved in amino acid biosynthesis, DNA replication, recombination and repair pathways, and transcriptional regulators. In efforts to identify gain of function mutations, methyl nitro-nitrosoguanidine mutagenesis was performed on a *sigS-lacZ* reporter fusion strain. From this a number of clones displaying *sigS* upregulation were subject to whole genome sequencing, leading to the identification of the lactose phosphotransferase repressor, *lacR*, and the membrane histidine kinase, *kdpD*, as central regulators of *sigS* expression. Again using EMSAs we determined that LacR is an indirect regulator of *sigS* expression, while the response regulator, KdpE, directly binds to the promoter region of *sigS*.

**Conclusions:**

Collectively, our work suggests a complex regulatory network exists in *S. aureus* that modulates expression of the ECF sigma factor, σ^S^.

**Electronic supplementary material:**

The online version of this article (doi:10.1186/s12866-014-0280-9) contains supplementary material, which is available to authorized users.

## Background

*Staphylococcus aureus* is an exceedingly virulent and successful pathogen, capable of causing a wide range of infections, from relatively benign skin lesions to life threatening septicemia. With an overwhelming ability to adapt to its environment, *S. aureus* has become the most common cause of both hospital and community acquired infections, and is believed to be the leading cause of death by a single infectious agent in the United States [1,2]. The threat posed by this organism to human health is further heightened by the rapid and continued emergence of multi-drug resistant isolates [1-4]. The success of *S. aureus* as a pathogen can, in part, be attributed to its many immune-evasion strategies, mediated by secreted and surface-associated virulence determinants. These virulence determinants have multi-faceted roles, allowing for adhesion, immune subversion and dissemination.

The regulation of gene expression is a fundamental property to all living systems that allows them to adapt and respond to different environmental conditions. Regulation can occur at the level of transcription, translation and post-translation, with the primary point being at the level of transcription. In bacteria, transcription is catalyzed by RNA polymerase (RNAP) whose promoter recognition and selectivity is influenced by a variety of transcription factors, with the most important group being sigma factors. In order for prokaryotes to initiate transcription, a sigma factor is required to associate with core RNAP. Once associated, sigma factors play a role in promoter recognition, and promoter melting to form the transcriptional open complex. All prokaryotes have a primary sigma factor, σ^A^, which mediates transcription of the majority of genes, including those with housekeeping functions (e.g. replication machinery, cell division). In addition to this, alternative sigma factors exist, which control subsets of genes that are involved in specialized cellular functions or stress responses (e.g. oxidative stress, heat shock, *etc.*) [5]. In 1994, Lonetto *et al*. described a subfamily of alternative sigma factors known as extracytoplasmic function (ECF) sigma factors; and over the past two decades there has been a large number of these elements identified [5-7]. Indeed, ECF sigma factors now represent the most numerate sub-family of these enzymes, with many bacteria possessing multiple such factors; so that this group outnumbers all other types of alternative sigma factors combined [5]. In addition to their growing number, their role in virulence is become increasingly apparent. In *Pseudomonas aeruginosa,* ECF sigma factors play an important role in iron uptake pathways, alginate secretion, and the expression of virulence factors, all of which contribute to pathogenesis. With such a complex life style, it would not be surprising if several ECF sigma factors were present in the genome of *S. aureus*. However, to date, only one has been identified, σ^S^, which forms the basis of this work [7].

Our group has previously shown that purified σ^S^ is able to bind core-RNAP and initiate transcription from its own promoter. A *sigS* mutant was found to be more sensitive to lysis by Triton X-100, and is outcompeted in long-term growth experiments by the parent under both standard conditions and in the presence of chemical stressors. Using a murine model of septic arthritis, it was also found that *sigS* is required for full virulence of *S. aureus* [7]. More recently, our group demonstrated that *sigS* expression is not observed under standard conditions except in the highly mutated strain, RN4220 [8]. However, *sigS* expression is induced in the presence of a number of chemical stressors that include DNA damaging and cell wall targeting agents. In addition, this element is upregulated upon challenge by components of the immune system, and during phagocytosis by murine macrophage-like cells [8].

The control of alternative sigma factor expression has been increasingly documented, with a number of novel genetic regulatory mechanisms identified [9,10]. A recent study regarding *sigH* in *S. aureus*, demonstrates that the expression of this element can only be induced by a chromosomal gene duplication rearrangement that occurs spontaneously, generating a new chimeric *sigH* gene driven by the promoters of either *nusG* or *rplK* [9]. A second, post-transcriptional mechanism of regulation also occurs, through an upstream inverted repeat that suppresses the translation of σ^H^ protein [9]. With regards to the regulation of ECF sigma factors, a number of studies have described unique mechanisms of regulation, including for an ECF sigma factor that is closely related to σ^S^, σ^M^ of *B. subtilis*. In a recent study it was shown that inactivation of a conserved membrane protein, *yfhO*, leads to an increase in activity of a putative bactoprenol glycosyltransferase. This subsequently leads to activation of σ^M^ [10], as the shortage of available bactoprenol impacts cell envelope integrity, which σ^M^ is believed to play a role in protecting [11].

In this study we expand on our previous work exploring the environmental induction of *sigS* expression in *S. aureus*, by focusing at the genetic level, so as to understand the regulatory circuits involved in controlling the expression of this gene.

## Methods

### Bacterial strains and growth conditions

*S. aureus* and *E. coli* strains, along with plasmids and primers, are listed in Table 1. *E. coli* was grown in Luria-Bertani (LB) medium at 37°C with shaking at 250 rpm. *S. aureus* was grown in Tryptic Soy Broth (TSB) at 37°C with shaking at 250 rpm, unless indicated otherwise. Synchronized cultures were obtained as previously describe [7]. When required, antibiotics were added at the following concentrations: ampicillin 100 μg ml^−1^, tetracycline 5 μg ml^−1^, erythromycin 5 μg ml^−1^, lincomycin 25 μg ml^−1^, and 0.25 mM cadmium chloride. Where specified, agar plates contained the DNA damaging agent methyl methanesulfonate at a concentration of 0.25 mM.

**Table 1:** **Strains, plasmids and primers used in this study**

**Strain, plasmid or primer**	**Genotype or description**	**Reference or source**
*E. coli*		
DH5α	φ80 Δ(*lacZ*)*M15* Δ(*argF-lac*)*U169 endA1 recA1*	[12]
	hsdR17 (r_K_^**−**^m_K_^+^) deoR thi-1 supE44 gyrA96 relA1	
*S. aureus*		
RN4220	Restriction deficient transformation recipient	Lab stocks
8325–4	Wild-Type Laboratory Strain *rsbU* mutant	Lab stocks
SH1000	Wild-Type Laboratory Strain *rsbU* functional	[13]
LES57	SH1000 pAZ106::*sigS*-*lacZ sigS* ^*+*^	7
HKM08	8325–4 pAZ106::*sigS*-*lacZ sigS* ^*+*^	This study
HKM15	LES57 isolate subject to random mutagenesis using nitrosoguanidine	This study
HKM16	LES57 isolate subject to random mutagenesis using nitrosoguanidine	This study
HKM17	8325–4 pSC-A::*tet*::*sigS-lacZ sigS* ^*+*^	This study
HKM18	8325–4 pSC-A::*tet*::*sigS-lacZ sigS* ^*+*^ pRN3208	This study
Plasmids		
pSC-A	TA clone vector lacking Gram-positive origin of replication	Strata clone
pRN3208	TS shuttle vector harboring Tn*551*	15
pDG1515	Tetracycline resistance antibiotic cassette in Bluescript KS(+) (Amp^r^)	[14]
pLES205	pAZ106 containing a 1. 4 kb *sigS* fragment	7
pHKM4	pSC-A containing the *sigS-lacZ* fragment from pLES205	This study
pHKM5	pSC-A containing a *tet* cassette and *sigS-lacZ* fragment from pLES205	This study
Primers^1^		
OL281	ACT *GGA TCC* CAG TTG CAG ATG CAT CTC TCC	
OL388	GTT GTC TGA ATA AAT CGA TAA GG	
OL522	ATG *TCT AGA* GAG TAA TGC TAA CAT AGC	
OL523	ATG *TCT AGA* CCC AAA GTT GAT CCC TTA ACG	
OL909	ATG *CTG CAG* CAG GAC CCA ACG CTG CCC GAG	
OL1039	[Biotin]CGT GCC TTC AAT TTG ACC ATC ACG	
OL1184	AGC CGA CCT GAG AGG GTG A	
OL1185	TCT GGA CCG TGT CTC AGT TCC	
OL1471	TTT ATG GTA CCA TTT CAT TTT CCT GCT TTT TC	
OL1562	[biotin]GTA ATC CAT TGT TAC CTC CCG	
OL1563	[biotin] GTG GTG TTT GTT GTA TAC GTC	
OL1568	CGA TTA CGC AAA TGA ATG	
OL1569	CAA GTA GTC ATT CTC CAA G	
OL1942	GTA ATC CAT TGT TAC CTC CCG	
OL1943	GTG GTG TTT GTT GTA TAC GTC	
OL2967	[biotin]GGC TTT CAA TTT GAT TAC GTT T	
OL2968	[biotin]CTA AAT TAA AAG TAT AAC TGC ATT G	
OL2979	[biotin]GTA CAC CTC ATA TTA CGA CTT TTT C	
OL2980	[biotin]CAT TAG TGA GAA TCA TTG TCA ATT AG	
OL3012	[biotin]CCA TGA TAA CCC TCA CTT AAT ATA	
OL3013	[biotin]GAC ATA ACC TTC ACC TCG ATA GCA	
OL3014	CCA TGA TAA CCC TCA CTT AAT ATA	
OL3015	GAC ATA ACC TTC ACC TCG ATA GCA	
OL3016	[biotin]GCA TCT GAC ACA CAA GTA TTT GTG TTG	
OL3017	[biotin]CCG AGT CTG TCT TTA ACA CTG	
OL3018	GCA TCT GAC ACA CAA GTA TTT GTG TTG	
OL3019	CCG AGT CTG TCT TTA ACA CTG	

^1^Restriction sites are italicized.

### Construction of a tetracycline marked *sigS-lacZ* strain

The *sigS* promoter region and *lacZ* gene were amplified from a previously constructed reporter fusion strain using primers OL281/OL909 (Table 1) [7]. This fragment was subsequently TA cloned into pSC-A using StrataClone PCR Cloning Kit as described by the manufacturer’s instructions (Agilent Technologies, Santa Clara, CA). In order to generate a tetracycline marked fusion, primer pair OL522/OL523 was used to amplify the *tet* resistance cassette from pDG1515 then digested with XbaI and ligated into similarly cut pSC-A containing the *sigS*-*lacZ* fragment. The resulting plasmid, pHKM5, was then transformed into *S. aureus* RN4220 and integrants were confirmed by PCR analysis using a forward primer that is specific to *sigS*, and a reverse primer that is specific to *lacZ*. A representative clone was then used to transfer the fusion into *S. aureus* 8325*–*4 by φ11-mediated transduction. This produced strain HKM17, which was confirmed by similar PCR analysis.

### Identification of proteins that bind to the *sigS* promoter using a biotin pull down assay

A 553 base pair fragment, which contains the *sigS* promoter sequence, was amplified by PCR using primers OL388 and OL1039, with the upstream primer containing a biotinylated tag. PCR products were separated on a 1% agarose gel, and purified using the QIAquick gel extraction kit (Qiagen) according to the manufacturer’s protocol. Overnight cultures of *S. aureus* RN4220 were subcultured into 100 ml of fresh TSB and grown for 3 hours, before being standardized to an OD_600_ of 0.05 and allowed to grow for 2 hours. Cells were harvested by centrifugation and washed in 10 ml of TE buffer (10 mM Tris–HCl, 1 mM EDTA, pH 8), before being resuspended in 1 ml of phosphate buffered saline. Cytoplasmic proteins were then extracted as previously described [15], before being stored at −80°C.

Dynabeads M-280 streptavidin magnetic beads (Life Technologies) were transferred to a 1.5 ml centrifuge tube and placed on a magnetic rack for 2 minutes before being washed twice with 1 ml PBS containing 0.2% Triton X-100. Beads were then incubated with either the *sigS* promoter region, *nsaRS* promoter region (control DNA fragment) or nuclease free water (negative control) at 25°C for 15 minutes. Following this, beads were again washed twice with 1 ml PBS and 0.2% Triton X-100. In order to prevent promiscuous protein binding to any unbound streptavidin beads, the beads/DNA complex was then incubated in the presence of 10 μg ml^−1^ biotin for 15 minutes at 25°C, followed by two washes with PBS and Triton X-100. RN4220 protein lysates were thawed on ice before the addition of 2 μg of poly(dI-dC), to act as a DNA competitor. Lysates were then added to the beads/DNA complex and incubated for 30 minutes at 25°C. Samples were subsequently washed 5 times with 1 ml of PBS and 0.2%Triton X-100, before being eluted with 50 μl of nuclease free water. Samples were run on an SDS-PAGE gel and silver-stained to visualize proteins, before being subjected to in-gel trypsin digestion as previously described [16], and identification by mass-spectrometric analysis.

### Transposon mutagenesis and DNA sequencing of insertion sites

Transposon mutagenesis was carried out as previously described [17]. Briefly, a plasmid harboring the Tn*551* transposon, pRN3208, was introduced into an 8325–4 *sigS*-*lacZ* strain by φ11-mediated transduction. The resulting strain, HKM18, was initially grown overnight at 30°C on a TSA plate containing 0.25 mM CdCl_2_, 5 μg ml^−1^ erythromycin, 5 μg ml^−1^ tetracycline (CET). A flask of 100 ml TSB_CET_ was inoculated from this plate and grown overnight at 30°C. A 5 ml volume from the overnight was washed once with TSB and resuspended in 100 ml TSB containing erythromycin, and grown at 43°C. After 4 hours, another 5 ml of culture was transferred to 100 ml TSB containing erythromycin and allowed to grow at 43°C until the OD_600_ reached 1.5 [18]. One ml aliquots were then collected via centrifugation, resuspended in TSB glycerol (20% v/v) and stored at −80°C for future analysis. The CFU ml^−1^ and insertion rate of the library was determined via serial dilution and plating on TSA containing erythromycin and TSA containing cadmium chloride (0.25 mM). The CFU ml^−1^ was determined to be 5.9 × 10^9^ and the insertion rate was 99.4%. For analysis, glycerol stocks were plated onto TSA containing erythromycin and 5-bromo-4-chloro-2-indolyl-β-D-galactopyranoside (X-GAL) at approximately 200 colonies per plate and analyzed for the appearance of blue colonies, indicating *sigS* upregulation. Isolates with blue coloration were collected, and used to prepare φ11 phage lysates. To confirm linkage of the phenotype with the transposon, mutations were transduced into a clean 8325–4 *sigS*-*lacZ* strain and again inspected for blue coloration. Any strain resulting in a positive result during this secondary screen was stored as a glycerol at −80°C, and genomic DNA prepared utilizing a QIAGEN DNeasy Tissue Kit, as per the manufacturer’s instructions. The identification of insertion sites was performed utilizing single-strand DNA sequencing with primer OL1130, specific to Tn*551*. This approach allows for the sequencing of a small fragment of Tn*551*, and approximately 500 bp of flanking genomic DNA outside of the transposon. Insertion sites were then identified utilizing NCBI BLAST analysis.

In an effort to identify genes that have a positive impact on *sigS* expression, the previously generated 8325–4 *sigS*-*lacZ* transposon library was also plated on agar that contains 0.25 mM of the DNA damaging agent methyl methanesulfonate (MMS) and X-GAL. MMS was used because previous studies by our lab have shown that expression of *sigS* is induced by this agent [8]. Glycerol stocks of the transposon library were thawed, and plated at approximately 200 colonies per plate, before being analyzed for clones that lacked a blue coloration. Such colonies were isolated, subject to secondary screening, and the insertion site of transposon insertion determined, as described above.

### β-Galactosidase assay

Levels of β-galactosidase activity were measured as described previously [19]. The results presented herein are the average of three independent replicates.

### Generation of 8325–4 *sigS*-*lacZ* NTML strains

The Nebraska Transposon Mutant Library (NTML) was acquired from the Network on Antimicrobial Resistant in *Staphylococcus aureus (*NARSA). Those mutations identified in the Tn*551* screens leading to altered *sigS* expression were transduced from relevant NTML clones into the 8325–4 *sigS*-*lacZ* strain using φ11. Each mutant was then validated using a gene specific primer (see Additional file 1: Table S1) and a primer specific to the *bursa aurealis* transposon (OL1471, Table 1).

### Nitronitrosoguanidine mutagenesis

An overnight culture of *S. aureus* SH1000 *sigS*-*lacZ* was synchronized and allowed to grow to mid-exponential phase, before 50 μg ml^−1^ N-methyl-N'-nitro-N-nitrosoguanidine (MNNG) was added, and allowed to incubate at 37°C for 60 min [20]. Cells were then collected via centrifugation at 3,500 rpm, washed, resuspended in TSB and allowed to recover at 37°C for 2 h [21,22]. Following the recovery period, 1 ml aliquots were collected via centrifugation and stored in TSB glycerol at ^−^80°C. The CFU ml^−1^ for cultures was determined both prior to MNNG exposure, and after, in order to determine survival rates (≥50% required). For analysis, the frozen samples were allowed to thaw, before being serially diluted in sterile PBS and plated on to TSA containing X-GAL. Plates were incubated overnight, before being inspected for the appearance of blue colonies, indicating increased *sigS* expression as a result of single nucleotide polymorphisms (SNPs) caused by the MNNG.

### Whole genome sequencing and data analysis

Whole genome sequencing was performed using an Ion Torrent Personal Genome Machine (PGM, Life Technologies) according to manufacturer’s protocols, as described by us previously [23]. Genomic DNA was extracted as described above and 1 μg of total DNA was fragmented using The Ion Xpress™ Plus Fragment Library Kit (Life Technologies). DNA was size selected using the E-gel system (Invitrogen) based upon the manufacturer’s recommendations for 200 base pair sequencing protocols. The size selected DNA was used to generate templated Ion Sphere Particles (ISPs) using an Ion OneTouch™ 200 Template Kit and an Ion OneTouch instrument. The resulting ISPs were sequenced on an IonTorrent 318 chip using a PGM™ 200 Sequencing Kit (Life Technologies). Sequences generated were exported in the .sff file format and analyzed using the CLC Genomics WorkBench software package. The previously sequenced genome of closely related strain NCTC 8325 was downloaded from the NCBI database (http://www.ncbi.nlm.nih.gov/genome/154?project_id=57795) and used as a reference to assemble each of the sequenced strains. Variant calling was performed within CLC Genomics WorkBench, and polymorphisms were selected on the basis that they were present in >95% of reads, with a minimum coverage of at least 10 reads.

### Quantitative real-time PCR

Quantitative real-time PCR was performed as previously described using primers specific to *sigS* [8,24]. The 16S rRNA gene was used as a control, as previously described [25].

### Purification of recombinant DNA binding proteins

The coding regions of *lacR*, *cymR,* and *kdpE* were cloned into pET24d + as previously described [7,26], before being transferred to *E. coli* BL21(DE3)pLysS. Cloning the coding region of the specified genes into pET24d + results in the addition of a C-terminal histidine tag. Expression of *lacR*, *cymR* and *kdpE* was induced with the addition of 1 mM isopropyl-1-thio-β-D-galactopyranoside (IPTG) to a growing 2 liter culture (OD_600_ = 0.6), before being allowed to grow for 5 hours at 37°C. Cells were harvested, resuspended in lysis buffer (50 mM NaH_2_PO_4_, 300 mM NaCl, 10 mM imidazole, pH 8.0) containing 1 mg ml^−1^ lysozyme, and incubated at 37°C for 1 hour, followed by sonication. All crude protein extracts were then applied to a nickel-nitrilotriacetic acid (Ni-NTA) metal- affinity matrix followed by washing with wash buffer (50 mM NaH_2_PO_4_, 300 mM NaCl, 20 mM imidazole, pH 8.0). Proteins were eluted using elution buffer (50 mM NaH_2_PO_4_, 300 mM NaCl, 250 mM imidazole, pH 8.0). Following elution, purified proteins were dialyzed for 48 hours against a dialysis buffer (50 mM NaH_2_PO_4_, 300 mM NaCl, pH 8.0). The resulting C-terminally histidine tagged, purified proteins were stored in dialysis buffer supplemented with 10% glycerol and kept at −20°C.

### Electrophoretic mobility shift assay

The promoter of *sigS* was amplifying using primers OL1562 and OL1563 and chromosomal DNA isolated from *S. aureus* USA300 HOU. As a positive control for purified LacR, the promoter fragment of the *lac* operon was amplified using primers OL2967 and OL2968. It has previously been shown that LacR binds to and represses transcription of the *lac* operon [27]. For CymR, the promoter of *mccAB* was amplified using primers OL3016 and OL3017. CymR has previously been shown to directly bind and repress transcription of this operon [28]. The positive control for the response regulator KdpE was the promoter region of *kdpFABC*, which was amplified using OL3012 and 3013. KdpE has previously been shown to bind and repress transcription from *kdpFABC* [29]. The PCR products were biotinylated at the 5’ and 3’ ends, and shift assays were performed using LightShift Chemiluminescent EMSA Kit (Pierce) according to the manufactures protocol. Briefly, labelled promoter fragments were incubated at 25°C for 20 minutes with various amounts of purified proteins in 20 μl reaction buffer (1× binding buffer, 2.5% glycerol, 50 ng ml^−1^ poly dI-dC, and 0.05% NP-40). The binding buffer used for LacR contained 20% glycerol, 5 mM MgCl_2_, 25 mM EDTA, 2.5 mM DTT, 250 mM NaCl, 50 mM Tris–HCl pH 7.5, and 0.25 mg/ml poly dI-dC. The CymR and KdpE binding buffer contained 20 mM Tris–HCl pH 7.5, 10 mM MgCl_2_, 100 mM KCl, 10 mM CaCl_2_, 1 mM EDTA, and 10% glycerol.

## Results

### The identification of proteins that modulate *sigS* expression

**Figure 1:**
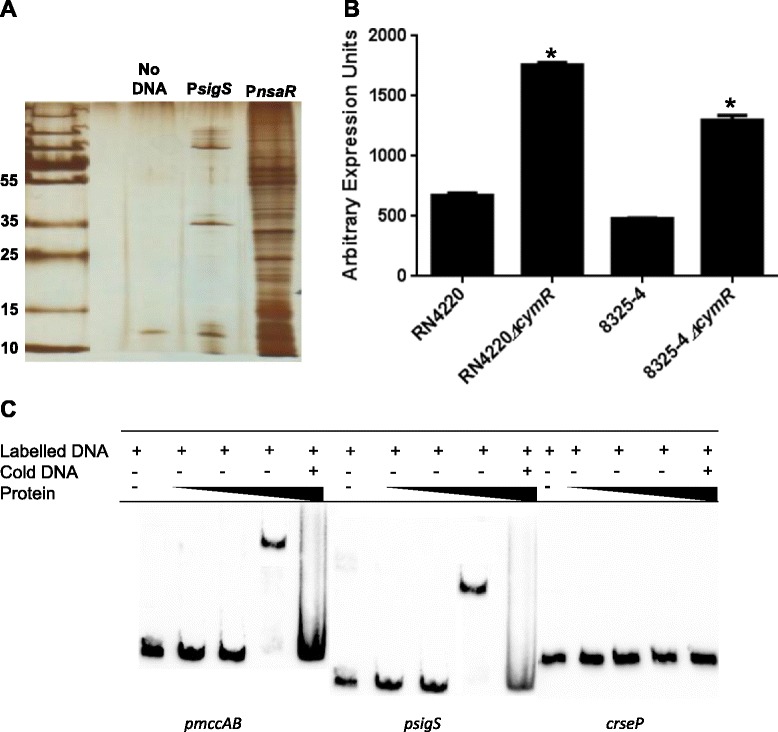
**CymR is a direct repressor of transcription from the** ***sigS*** **promoter. (A)** A pull down assay was performed using crude protein lysates harvested from RN4220 at hour 2 and a biotinylated *sigS* promoter DNA probe. The identity of protein bands was determined by LC/MS analysis. **(B)** qPCR analysis of *sigS* expression in a *cymR* mutant compared to its respective parental strain, in the 8325–4 and RN4220 backgrounds. Error bars are shown as ± SEM, * = p <0.05 using a Student’s *t*-test. **(C)** An electrophoretic mobility shift assay was performed using purified CymR, and the promoter region of *mccAB* (positive control), the promoter region of *sigS* (test), and an intergenic region from the *rseP* gene (*rseP*; negative control). CymR was added at increasing concentrations of 0.01, 0.1 and 1 μM in all panels.

Previous studies on *sigS* expression by our group have demonstrated significant variability across different wild-type strains. This has led us to hypothesize in the past that there may be regulatory circuits that exists in *S. aureus* to control the expression of this gene [8]. As such, in order to identify direct regulators of *sigS* expression, a pull down assay was performed using a biotinylated *sigS* promoter fragment immobilized on streptavidin magnetic beads. Protein lysates from strain RN4220 were utilized in these assays, as our group previously demonstrated *sigS* expression to be highest in this background under standard conditions [8]. Protein lysates from RN4220 grown under standard conditions for 2 h were incubated with the biotinylated *sigS* promoter fragment. This time point was selected in order to identify modulators of *sigS* that effect transcript levels as the bacteria transition from minimal *sigS* expression (2 h) to maximal expression by hour 3 [8]. Proteins that were bound to the promoter region were harvested, and separated by SDS PAGE analysis followed by silver staining (Figure 1A). We observed that while there were no proteins detected in our no DNA control lane, several distinct bands were detected in the *sigS* test lanes. For our control promoter (*nsaXRS*), we observed a number of proteins bound to this fragment, which likely results from the fact that this locus is very highly expressed, and subject to complex, multifactorial regulation [30,31]. The identity of proteins bound to the *sigS* promoter was then determined using mass-spectrometric analysis, identifying 21 different factors (Additional file 1: Table S2). Whilst a number of proteins were identified with known or predicted DNA-binding roles (including DNA replication and transcription, stress proteins and exonucleases), only one was a known transcriptional regulator: the cysteine biosynthesis pathway transcriptional regulator, CymR. As this did not appear in either of the control samples, we next set out to determine if CymR had a measurable impact on *sigS* expression using qPCR and mutant strains deficient in the *cymR* gene. This analysis was performed in strains RN4220 and 8325–4: the former because this is the background where the identification was made and the latter as we have previously shown that this strain is highly sensitive to *sigS* modulation [8]. For RN4220 and 8325–4, RNA was harvested at 3 and 5 hours, respectively, as this is the time for each strain when *sigS* is maximally expressed [8]. Upon analysis, we observed a 2.7- and 2.6-fold increase, respectively, in *sigS* expression in the 8325–4 and RN4220 *cymR* mutants compared with the wild-type strains (Figure 1B). This would seem to indicate that the CymR protein plays a role in the negative regulation of *sigS* expression in *S. aureus*. To determine if this effect is indeed direct, we sought to recapitulate the binding of this protein to the *sigS* promoter *in vitro* using electrophoretic mobility shift assays (EMSA) (Figure 1C). The promoter region of *mccAB* was used as a positive control because Soutourina *et al.* have previously shown that CymR directly binds to this region [28]. When the *mccAB* promoter fragment was incubated with increasing amounts of CymR, a clear shift is observed at higher concentrations of protein. Interestingly, parallel experiments with the *sigS* promoter fragment revealed similar shifts, which would indicate that CymR is a direct regulator of *sigS* expression. In order to demonstrate that this effect was specific and not a result of promiscuity, we performed EMSAs using a biotinylated DNA fragment that was amplified from the coding region of the gene *rseP*. In these studies we did not observe any shift of the DNA, validating that the binding observed for *sigS* is indeed specific, and that CymR directly regulates, and represses, transcription from the *sigS* promoter.

### A global screen to identify genes that negatively regulate *sigS* expression

To assess whether additional regulators of *sigS* expression exist in *S. aureus* beyond CymR, we next used a global genetic approach to identify modulating factors. Therefore, we employed transposon mutagenesis screen in conjunction with an 8325–4 *sigS*-*lacZ* reporter fusion strain. This background was chosen because our previous works have shown that 8325–4 is the most sensitive wild-type strain to *sigS* modulation, and that information gained using this strain is conserved amongst other *S. aureus* wild-types [8]. As such, we screened >10,000 clones from our transposon library, and identified 123 that had increased *sigS* expression. A secondary screen was performed on these mutations, by transducing them into a clean 8325–4 *sigS*-*lacZ* fusion background. This was to ensure that the increase in expression is due to direct gene disruption by Tn*551*, rather than the result of SNPs that may have accumulated during construction of our library. We determined that 96 mutants from our secondary screen recapitulated the increase in *sigS* expression on media containing X-GAL. Upon sequence analysis, we identified 58 unique insertion sites, with 49 found to be in coding regions, and a further 9 found to be intergenic between open reading frames (Additional file 1: Table S3).

**Figure 2:**
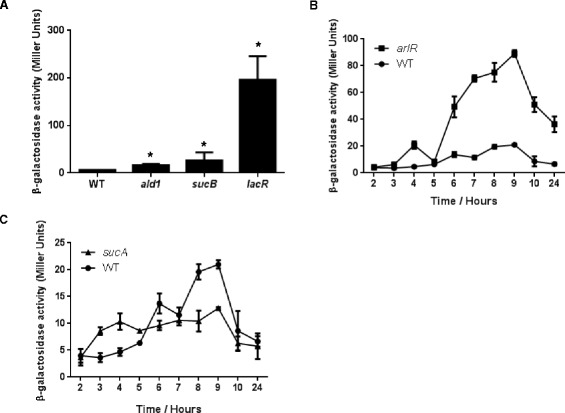
**Transcriptional profiling of** ***sigS*** **in transposon mutants found to negatively effect expression. (A, B, and C)** Mutant strains bearing a *sigS-lacZ* fusion were grown in TSB at 37°C and samples withdrawn at the times specified. β-Galactosidase activity was measured using 4-MUG as a substrate to determine *sigS* expression levels. Assays were performed on duplicate samples and the values averaged. The results presented are from three independent experiments. Error bars are shown as ± SEM. Significance was determined by a Student *t* test; *indicates a p value of <0.05.

Analysis of transposon insertion sites revealed many were clustered in one particular region of the genome (SACOL1411 to SACOL1490). We have previously identified this region as a hot-spot for Tn*551* insertion [17], and therefore sought to validate our findings further to ensure they specifically related to *sigS* expression. To achieve this, we made use of the Nebraska Transposon Mutant Library (NTML), a collection of *bursa aurealis* transposon mutants in almost all non-essential *S. aureus* gene [32]. Of the 49 identified insertions from our screen that were in coding regions, 40 had NTML mutants available (Additional file 1: Table S3). Therefore, each of the NTML insertions for these 40 genes were separately introduced into our 8325–4 reporter strain. In order to determine if the NTML mutants recapitulated our *sigS* expression findings from the Tn*551* screen, all mutants were first analyzed using a plate based assay. Upon analysis, 20 of the transduced NTML mutants were blue when plated on media containing X-GAL (Table 2), whilst the remaining 20 did not reproduce the findings of their counterpart Tn*551* insertions. β-galactosidase assays were then performed on the NTML mutants that tested positive, to specifically quantify changes in *sigS* expression. As such, NTML reporter fusion strains were grown for 5 h in TSB, as we have previously shown this to be the window of peak *sigS* expression in strain 8325–4 [8]. Interestingly, only three mutants were found to have increased *sigS* expression at this time point (Figure 2A): insertions in *ald1* (an alanine dehydrogenase, 2.6-fold), *lacR* (the lactose phosphotransferase system repressor, 31-fold) and *sucB* (dihydroipoamide succinyltransferase, 4.2-fold). For the 17 mutants that did not demonstrate increased *sigS* expression at this time point, we next performed transcriptional profiling every hour over a 10 hour time course. In doing so, we observed increased *sigS* expression for an additional 2 mutants (Figure 2B and C): insertions in *arlR* (a DNA-binding response regulator), and *sucA* (2-oxoglutarate dehydrogenase E1 component). For *arlR* we observed an increase in *sigS* expression at all hours assayed (maximal change =6.1 –fold at 9 h) except at 5 h; whilst for *sucA*, a gene transcribed upstream of *sucB*, we observed a 2.2 fold increase at hour 4. With regards to the remaining 15 transposon mutants, we did not see an increase in *sigS* expression over the 10 hour time course. However, we did observe a blue coloration when plated on media containing X-GAL, which does not occur with wild-type 8325–4 *sigS*-*lacZ* fusions. As such, the increase in *sigS* expression observed for these strains either takes place deeper into stationary phase than assessed herein; or as a result of growth on a solid surface compared with liquid culture.

**Table 2:** **Transposon insertions resulting in increased expression of** ***sigS***

**Accession number**	**NE number** ^**a**^	**Tn** ***551*** **insertion site**	**Gene**	**Hits** ^**b**^	**Unique** ^**c**^
**DNA metabolism: DNA replication, recombination and repair**					
SACOL0566	NE544	Nucleoside permease	*nupC*	1	1
**Regulators**					
SACOL0513	NE1883	Transcriptional regulatory protein	*glcT*	1	1
SACOL1451	NE1684	Response regulator	*arlR*	1	1
SACOL1436	NE9	Modulator of SarA	*msa*	1	1
SACOL2086	NE1218	Transcriptional regulator, TenA family	*tenA*	1	1
SACOL2188	NE436	Lactose phosphotransferase system repressor	*lacR*	5	1
**Cell envelope associated**					
SACOL1472	NE1	Cell wall associated fibronectin-binding protein	*ebh*	6	2
**Transporters**					
SACOL0684	NE1292	Na+/H + antiporter, MnhE component, putative		2	1
SACOL1319	NE1580	Glycerol uptake facilitator protein		5	1
SACOL1392	NE142	Sodium:alanine symporter family protein		3	1
SACOL1414	NE1609	Peptide ABC transporter, ATP-binding protein		4	1
**Amino acid biosynthesis**					
SACOL0168	NE595	Glutamate N-acetyltransferase/amino-acid acetyltransferase	*argJ*	2	1
SACOL1349	NE809	Threonine aldolase		1	1
SACOL1448	NE1391	Dihydroipoamide succinyltransferase	*sucB*	1	1
SACOL1449	NE547	2-Oxoglutarate dehydrogenase, E1 component	*sucA*	3	1
SACOL1478	NE1136	Alanine dehydrogenase		1	1
SACOL2045	NE1177	Ketol-acid reductoisomerase		1	1
**Protein synthesis and modification**					
SACOL1369	NE1904	50S ribosomal protein l33		4	1
SACOL1480	NE582	Hypothetical protein (ribosome associated GTPase domain)		1	1
**Unknown function**					
SACOL1452	NE577	PAP2 family protein		1	1

^a^NE numbers are the identification number in the Nebraska Transposon Mutant Library.

^b^Hits refer to the total number of Tn*551* insertion sites identified for a particular gene.

^c^The unique number of insertions sites refers to those insertions that are a result of distinctive insertion events.

### Transposon mutagenesis to identify positive activators of *sigS* expression

**Figure 3:**
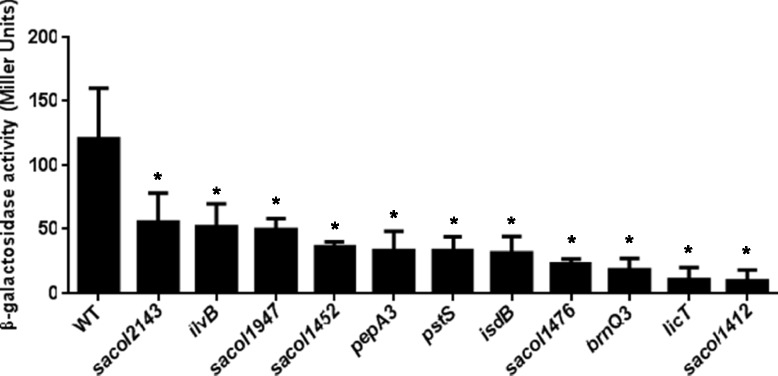
**Identification of positive regulators of** ***sigS*** **expression.** Mutant strains bearing a *sigS-lacZ* fusion were grown in TSB at 37°C and sampled after 5 h of growth. β-Galactosidase activity was measured using 4-MUG as a substrate to determine *sigS* expression levels. Assays were performed on duplicate samples and the values averaged. The results presented are from three independent experiments. Error bars are shown as ± SEM. Significance was determined using a Student *t* test; *indicates a p value of <0.05.

The advantage of using the 8325–4 *sigS-lacZ* fusion for transposon mutagenesis is that it can be performed in reverse, meaning that we can use a chemical that induces expression of *sigS* alongside X-GAL, and identify positive regulators by a lack of blue coloration upon transposon insertion. As such*,* media was prepared using 0.25 mM MMS and X-GAL, and our Tn*551* mutant library was again subject to screening. In total, we assessed >10,000 clones and identified 349 that had no observable *sigS* expression, determined by a lack of blue coloration of colonies. A secondary screen was performed with these strains, yielding 86 that recapitulated the phenotype. This relatively low number of clones that retained phenotype, compared to our original screen, is most likely due to point mutations caused through the use of the DNA damaging agent MMS. Upon sequencing, we identified 35 unique insertion sites (Additional file 1: Table S4), 24 of which occurred within genes, and 11 that were found to be intergenic. Interestingly, 12 insertions occurred within the known hotspot region, whilst a further 5 were identified in both screens (one of which failed to validate in our previous screen, and the other four failed to validate in this screen, see below). In an effort to define which of these elements legitimately influence *sigS* expression in a positive manner, we again made use of the NTML collection. Of the 24 insertions within ORFs, 21 mutants were available in the Nebraska Transposon Mutant Library. As such, each of these mutations was again transduced into a clean 8325–4 *sigS*-*lacZ* strain, and their effects on *sigS* expression validated by plating on media containing 0.25 mM MMS and X-GAL. Of the 21 mutations assayed, 10 of them were blue when plated on media containing MMS and X-GAL (Table 3), including the remaining 4 that were identified in both screens (thus excluding them from further study). The remaining 11 mutants had abrogated *sigS* expression, as expect, and were thus subject to transcription profiling in liquid culture in the presence of 0.25 mM MMS for validation (Figure 3). This time, all mutants tested resulted in decreased *sigS* expression after 5 h of growth, ranging from 2.2-fold (SACOL2143) to 12.2-fold (SACOL1412). Of the elements identified in this screen to positively influence *sigS* expression, there were insertions in genes whose products are involved in regulation, transport, protein synthesis and modification, amino acid biosynthesis, and cell envelope biosynthesis.

**Table 3:** **Transposon insertions resulting in decreased expression of** ***sigS***

**Accession number**	**NE number** ^**a**^	**Tn551 insertion site**	**Gene**	**Hits** ^**b**^	**Unique** ^**c**^
**Regulators**					
SACOL1393	NE1415	Transcriptional antiterminator, *licT* putative	*licT*	3	1
**Cell envelope associated**					
SACOL1138	NE1102	LPXTG cell wall surface anchor protein	*isdB*	1	1
**Transporters**					
SACOL1424	NE1459	Phosphate ABC transporter, phosphate binding protein		2	1
SACOL1443	NE44	Branched chain amino acid transport system II carrier protein	*brnQ3*	7	1
SACOL1476	NE211	Amino acid permease		1	1
**Amino acid biosynthesis**					
SACOL2043	NE1166	Acetolactate synthase, large subunit	*ilvB*	1	1
SACOL2154	NE134	Arginase	*rocF*	1	1
**Protein synthesis and modification**					
SACOL1402	NE1306	Glutamyl aminopeptidase, putative	*pepA3*	1	1
**Unknown Function**					
SACOL1452	NE577	PAP2 family protein		1	1
**Hypothetical proteins**					
SACOL1947	NE1706	Conserved hypothetical protein		3	1
SACOL2143	NE1104	Conserved hypothetical protein		2	1

^a^NE numbers are the identification number in the Nebraska Transposon Mutant Library.

^b^Hits refer to the total number of Tn*551* insertion sites identified for a particular gene.

^c^The unique number of insertions sites refers to those insertions that are a result of distinctive insertion events.

### Inducing *sigS* expression in strain SH1000

**Figure 4:**
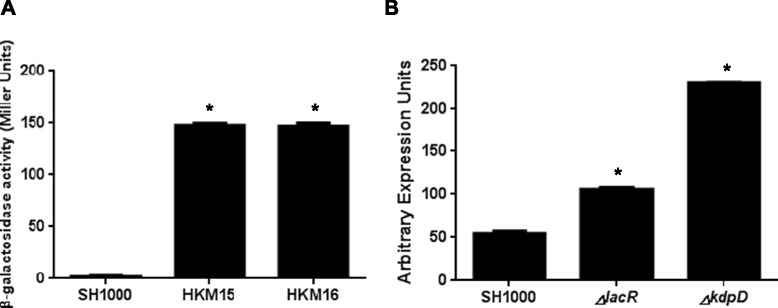
**Nitrosoguanidine mutagenesis identifies additional regulators of** ***sigS*** **expression. (A)** The SH1000 *sigS-lacZ* fusion strain was subjected to MNNG mutagenesis. Two resulting clones were selected for further analysis during growth in TSB at 37°C. HKM15 and HKM16 were grown along side the parent strain, SH1000, with samples collected at 5 hours. β-Galactosidase activity was measured using 4-MUG as a substrate to determine *sigS* expression levels. Assays were performed on duplicate samples and the values averaged. The results presented are from three independent experiments. Error bars are shown as ± SEM. **(B)** qPCR for *sigS* expression was performed on SH1000 strains containing a mutation in either *lacR* or *kdpD.* Error bars are shown as ± SEM. Significance was determined using a Student *t* test; *indicates a p value of <0.05.

*S. aureus* strain SH1000 is identical to 8325–4, apart from an 11 bp deletion in the σ^B^ controlling phosphatase, *rsbU*. Despite this similarity, SH1000 does not demonstrate detectable *sigS* expression during regular growth; an effect that appears to be more complex than mere σ^B^ mediated control [7,8]. Therefore, to explore whether *sigS* upregulation could be achieved in this strain, we next exposed our SH1000 *sigS-lacZ* fusion strain to the DNA mutagen, methyl nitro-nitrosoguanidine (MNNG). MNNG acts by adding alkyl groups to O^6^ of guanine and O^4^ of thymidine, which can lead to transition mutations. This has an advantage over transposon mediated technologies as it can assess both loss- and gain-of function, rather than only the former. Upon analysis, we identified 76 strains that had an increase in expression of *sigS* as determine by blue coloration when plated on media containing X-GAL. To quantitate the increased *sigS* expression observed on a plate, we selected two strains, HKM15 and HKM16, and subjected them to continuous growth analysis in liquid media (Figure 4A). We determined that both HKM15 and HKM16 resulted in a 75-fold increases in *sigS* expression after 5 h of growth, compared to the wild-type. To identify the specific mutations that result in these outcomes, we next subjected these two strains to whole genome sequencing, alongside the SH1000 parent, with resulting data analyzed using the CLC Genomic Workbench software. When one compares these two datasets, we found 9 mutations that were common to both strains (Table 4). Importantly, within this list are two known transcriptional regulators, LacR, which we have already identified in this study as influencing *sigS* expression, and KdpD, a membrane sensor histidine kinase. In order to evaluate the effect these mutations have on *sigS* expression, the relevant NTML mutations were transduced into SH1000, and qPCR profiling was performed (Figure 4B). We observed a 2.0-fold increase in *sigS* expression in the SH1000 *lacR* mutant, while we observed a 4.0-fold increase in *sigS* expression in SH1000 *kdpD*. This would indicate that lack of *lacR* and *kdpD* also have a role in regulating *sigS* expression.

**Table 4:** **SNPs common to both SH1000** ***sigS-lacZ*** **NTG generated mutants HKM15 and HKM16**

**Annotation**	**Amino acid change**		**Function**
	HKM15	HKM16	
SACOL0400	V342I	P307L	Transport SgaT, putative
SACOL1103	G64A	G64A	Pyruvate dehydrogenase complex E1 component
SACOL1213	E290K	A319V	Dihydroorotase
SACOL1472	A3615T	G2291D	Cell wall associated fibronectin-binding protein
SACOL1686	D388N	D388N	Histidyl-tRNA synthetase
SACOL2070	P257S	P257S	Sensor histidine kinase KdpD
SACOL2150	A1128T	V1818I	SasB protein (fmtB)
SACOL2176	A382V	A382V	Osmoprotectant transporter, BCCT family
SACOL2188	G8E	G112E	Lactose phosphotransferase system repressor

**Figure 5:**
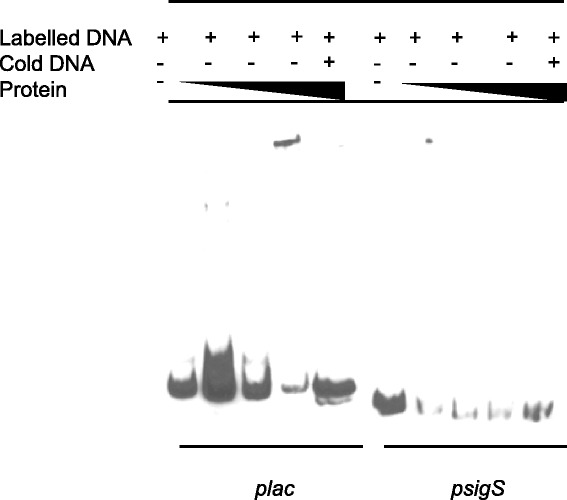
**LacR does not directly regulate** ***sigS*** **expression.** Electrophoretic mobility shift mobility assays were performed using purified LacR, the *lac* promoter region (positive control), and the promoter region of *sigS* (test). LacR was added at increasing concentrations of 0.01, 0.1 and 1 μM in all panels.

In an effort to determine if the effect on *sigS* expression is direct or indirect we performed EMSAs using purified LacR and KdpE. KdpE was used because it is the partner response regulator, phosphorylated by KdpD. With regards to LacR, the promoter region of the *lac* operon was used as positive control, as it has previously shown that LacR directly binds to this region [27]. As such, we incubated the *lac* promoter fragment with increasing amounts of LacR, and, as expected, observed a shift for the *lac* promoter fragment at higher protein concentrations (Figure 5). However, when the *sigS* promoter fragment was used, no such shift was observed. This suggests that, despite LacR clearly influencing *sigS* expression (it was identified in two of our screens), these effects appear to be indirect.

**Figure 6:**
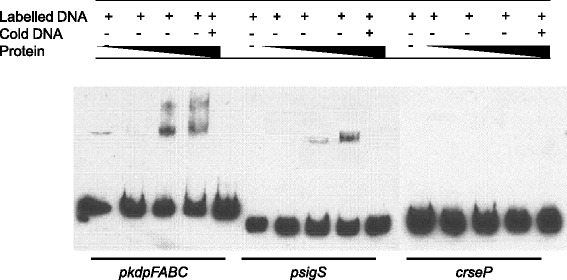
**KdpE directly regulates** ***sigS*** **expression.** Electrophoretic mobility shift mobility assays were performed using purified KdpE, the promoter region of *kdpFABC* (positive control), the promoter region of *sigS* (test), and an intergenic region from the *rseP* gene (negative control). KdpE was added at increasing concentrations of 0.1, 0.5 and 0.75 μM in all panels.

With regards to KdpE, we first used the promoter region of *kdpFABC* as a positive control, because it has previously been shown that KdpE directly binds this region [29]. As such, we incubated the *kdpFABC* promoter fragment with increasing amounts of KdpE, and detected a shift for this fragment at higher protein concentrations (Figure 6). Importantly, we also detected a shift for the *sigS* promoter fragment at similar concentrations, which would indicate that KdpE is a direct regulator of *sigS* expression. To determine if this is specific effect, and not the result of promiscuous binding by KdpE, we again used our internal fragment from the coding region of *rseP* as a negative control. In these studies we did not observe any shift of the DNA, validating that the binding observed for *sigS* is indeed specific, and that KdpDE directly regulates, and represses, transcription from the *sigS* promoter.

## Discussion

**Figure 7:**
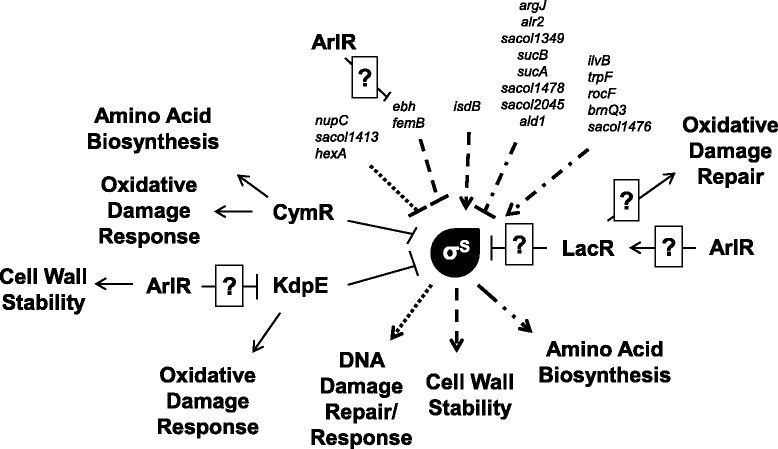
**Schematic representation of** ***sigS*** **regulation and role within the** ***S. aureus*** **cell.** Shown are genes identified in this study that either positively or negatively regulate *sigS* transcription; alongside known roles for σ^S^ from our previously published works. A correlation of input factors, to output functions, is denoted by similar line dashing.

The average bacterial genome is estimated to encode at least six ECF sigma factors [5,33]. *S. aureus* is a highly successful pathogen with complex regulatory circuits that, interestingly, encodes only one ECF sigma factor, σ^S^. This sigma factor has previously been shown to be important in the stress and virulence response of *S. aureus* [7]. In addition, we have also shown that *sigS* is differentially regulated across a number of *S. aureus* wild-type strains, which would indicate a network of regulation may exist, controlling the expression of this element. In earlier work by our group we have investigated the regulation of *sigS* by environmental signals in response to external stress; therefore in this study, we investigated the genetic mechanisms that regulate this transcription factor. We observed a number of findings that appear to correlate with our previous works regarding the function of σ^S^, detailed as follows (and in Figure 7).

To explore direct regulators of *sigS* expression, we used a biotin pull down assay in conjunction with the *sigS* promoter, and identified CymR as specifically binding to this region of DNA. Using qPCR we were able to demonstrate that *sigS* expression actually increased in a *cymR* mutant, indicating that this regulator is acting as a repressor. CymR belongs to the poorly characterized Rrf2 family of proteins, and has been shown to be the master regulator of cysteine metabolism in *S. aureus* [28,34]. Functionally, CymR binds directly to the promoter sequences of eight transcription units corresponding to 18 genes, and represses the transcription of elements involved in pathways leading to cysteine formation. Interestingly, in the presence of reactive oxygen species (ROS), it has been shown that there is derepression of several direct CymR targets [8,28,34]. We ourselves have previously shown that *sigS* expression is inducible during oxidative stress, and that *sigS* mutants are more sensitive to ROS. This would therefore suggest a link between cysteine metabolism, CymR, oxidative stress and *sigS* expression in *S. aureus*.

We identified further repressors of *sigS* expression in *S. aureus* using transposon mutagenesis as well as an MNNG based mutagenesis screen, with the most profound effects observed resulting from the inactivation of LacR. In *S. aureus*, the *lacR* gene encodes the transcriptional regulator for the lactose utilization system [27]. Because this gene encodes a transcriptional regulator, we were interested to see if the protein binds to the promoter region of *sigS*. Using EMSAs we determined that the regulatory affect we observe for *sigS* by LacR is indirect. It is unclear why the *S. aureus* LacR protein may function to regulate genes other than those involved in lactose utilization; however, it does share homology with *deoR* of *E. coli*, which is involved in repressing genes that are a part of the catabolism of deoxyribonucleosides. Interestingly, preliminary work by our group suggests that *sigS* may be involved in the *de novo* assembly of nucleotides (Miller and Shaw, unpublished observation). As such, it is possible that LacR may have retained an evolutionary role in nucleoside catabolism, which explains why it might connect to *sigS* expression in *S. aureus*. In addition, we also observed that when the response regulator *arlR* is disrupted there is an increase in *sigS* expression. This response regulator is involved in autolysis, adhesion and virulence of *S. aureus*. Interestingly, *arlR* mutants display an increase lysis when exposed to Triton X-100, which is a phenotype that we have previously described for *sigS* mutants [7,35]. Interestingly, an array performed on an *arlR* mutant indicates that this response regulator directly and/or indirectly controls expression of at least 114 genes. These include several genes identified in this study, such the *lacABCDFEG* operon, which is negatively regulated by *lacR*; *ebhAB*, which encodes an adhesin identified in our transposon screen, and the two component system *kdpDE*. In the context of this latter element we identified mutations in the sensor histidine kinase, *kdpD* as negatively influencing *sigS* expression. This gene is part of the two component system, KdpDE, which is involved in sensing and responding to potassium levels in the cell [36]. There has been an increase in evidence that demonstrates KdpDE is an adaptive regulator involved in the virulence and intracellular survival of pathogenic bacteria. Specifically, KdpDE has been shown to regulate genes involved in responding to oxidative stress, which we have previously shown *sigS* to be involved in [37].

We also identified a number of other genes as negatively influencing *sigS* expression that would appear to corroborate previous studies performed by our group. For example, we have shown that *sigS* expression is increased in the presence of DNA damaging agents [8]. Herein we identified insertions in genes that are involved in DNA replication, recombination and repair pathways as influencing the expression of *sigS*. Specifically, we identified a mutation in SACOL1413, which is a putative helicase demonstrating homology to Snf2 family of proteins that are thought to function in DNA damage repair [38]. Again, connected with this, we have demonstrated that cells deficient in *sigS* are more sensitive to a variety of DNA damaging agents, and that expression of *sigS* is similarly increased in the presence of such stressors. A mutation in the DNA mismatch repair protein, *hexA*, was also identified in our screen. HexA is a major component of the methyl mismatch repair system [39]. In *S. aureus,* disruption of *hexA* leads to a higher frequency of strains acquiring random SNPs [39]. Thus, it is plausible that, in the absence of *hexA*, the increased level of natural mutation would instigate a DNA-damage like-response, which seems to involve σ^S^ in the *S. aureus* cell. In addition, disruption of *nupC*, which encodes a nucleoside permease, increases expression of *sigS*. In the closely related bacteria *B. subtilis*, NupC is responsible for transporting all nucleosides other than hypoxanthine and guanine, which are then catabolized to purine and pyrimidine bases, and returned to the nucleotide pool for DNA biosynthesis [40]. This is of significant interest, as preliminary work by our group indicates that *sigS* mutants have severely diminished expression of nucleotide biosynthesis genes (Miller and Shaw, unpublished observation). This would suggest that in the absence of *nupC*, *sigS* expression is upregulated to aid nucleotide biosynthesis to replace those not being transported into the cell.

We also identified insertions in two genes that are involved in cell wall biosynthesis as having negative influence on *sigS* expression. This is of interest as we have previously reported that *sigS* expression is induced in the presence of antibiotics that targeting the cell wall, and that *sigS* mutants are more sensitive to this kind of stress [8]. The first of these two elements is *ebh*, a cell wall associated fibronectin-binding protein. Interestingly, cells deficient in *ebh* have been found to be more susceptible to lysis by Triton X-100, a phenotype that we also observed with a *sigS* mutant [8,41]. Ebh, which is localized over the entire cell surface, is the largest protein in *S. aureus*, and deletion of this factor from the cell has serious consequences to cell wall stability [41,42]. Thus, it is conceivable that in the absence of *ebh*, the expression of *sigS* would be increased in an effort to combat cell wall instability. Further to this, inactivation of *femB* also resulted in an increase in *sigS* expression. FemB is involved in cell wall biosynthesis, and *femB* mutants of *S. aureus* have been shown to have reduced glycine content of their peptidoglycan, leading to decreased cell wall stability and an increase in sensitivity to cell wall targeting antibiotics, such as β-lactams [43]. This data very closely mirrors our previous reports with σ^S^, explaining why cells deficient in *femB* might upregulate *sigS*.

There were also six insertions identified in the repressor screen for genes involved in amino acid biosynthesis. These include disruptions in the amino acid metabolism genes *asd* (aspartate-semialdehyde dehydrogenase), *ald1* (alanine dehydrogrenase), *alr2* (alanine racemase) and *argJ* (glutamate N-acetyltransferase). Connected to this, we have previously shown that *sigS* expression is increased in the presence of amino acid limiting media [8]. Moreover, in an earlier study, we have also demonstrated the importance for a functional σ^S^ during extended starvation [7]. As such, it appears possible that disruption in the normal flow of amino acids in the *S. aureus* cells necessitates that activity of σ^S^. We have also identified insertions in genes involved in alanine biosynthesis, including, SACOL1434. Further to this, in *B. subtilis, alr2* is responsible for the interconversion of L-isomer and D-isomer of alanine [44,45]. Interestingly, the ability for bacteria to synthesize D-alanine is essential for the biosynthesis of the cell wall in *B. subtilis* [45]. Therefore, the disruption of *alr2* could lead to an increase in *sigS* expression via two mechanisms: amino acid limitation and cell wall instability.

In the positive transposon screen, we again identified a number of mutations in genes that are involved in amino acid biosynthesis. These include *ilvB* (the large subunit of acetolactate synthase), *trpF* (phophoribosylanthranilate isomerase)*, brnQ3* (a branch-chain amino acid transporter), and SACOL1476 (a putative amino acid permease) that lead to a decrease in *sigS* expression. Thus, akin to that suggested above, it is plausible that without the ability to import and/or synthesize amino acids, *S. aureus* is unable upregulate *sigS* expression and repair the DNA damage caused by MMS. We also identified a mutation in *pepA3*, which encodes a putative glutamyl aminopeptidase. Glutamyl aminopeptidases are involved in cleaving glutamic and aspartic amino acids from the N-terminus of proteins. The *pepA* gene of *E. coli* encodes aminopeptidase A, a homohexameric multifunctional protein with aminopeptidase and DNA-binding activities, the latter being associated with mechanisms of transcriptional control and DNA recombination. Research has demonstrated that PepA is involved in the transcriptional regulation of the *carAB* operon, which encodes carbamoylphosphate synthetase; this enzyme catalyzes the ATP-dependent synthesis of carbamoyl phosphate from glutamine [46]. This process is the first committed step in pyrimidine and arginine biosynthesis. As we observed a decrease in *sigS* expression in a *pepA3* mutant, it may suggest a link between amino acid and pyrimidine biosynthesis, and *sigS*. Interestingly, we have also observed that *sigS* may be involved in both amino acid biosynthesis and pyrimidine biosynthesis ([8], Miller and Shaw unpublished observations).

In a global effort to identify regulators of *sigS* expression, two Tn*551* transposon screens were performed. A consideration with such approaches is that we have previously shown that Tn*551* has particular preference for a 108 kb hotspot region of the genome that extends from SACOL1411 to SACOL1490 [17]. In order to ensure that the findings generated in our study were specifically connected to transposon disruption, and not an artifact of transposition, we first set out to validate the role of genes identified in modulation of *sigS* expression. In our repressor transposon screen, we identified 51 unique insertion sites, 57% (29 mutants) of which fall within the known Tn*551* hotspot. In order to ensure that the *sigS* repression observed in these mutants directly connected to transposon insertion, we validated all Tn*551* insertions possible by parallel creation of mutants using the Nebraska Transposon Library. This is a collection of *bursa aurealis* transposon mutants in almost all non-essential *S. aureus* gene [32]. Of the 40 mutants available in the NTML for the repressor screen, only 20 retained phenotype when recreated using the *bursa aurealis* mutants, with only 9 of 17 from the hotspot region. In our transposon screen for activators of *sigS* expression, we identified 35 unique insertion sites, 51% (18 mutants) of which fell within the Tn*551* hotspot. The NTML contained 21 mutants to test for recapitulation, with 11 proving to retain phenotype upon recreating mutations. In this regard 11 of these mutants were originally in the hotspot, with 8 *bursa aurealis* transposon mutants validating alteration in *sigS* expression. Interestingly, we also identified 5 transposon mutants that were common to both screens. However, upon validation, none of the mutants retained phenotypes as both repressors and activators; with one of them proving to have only negative effects on *sigS* expression, whilst the remaining four were positive effectors. Overall, these findings suggest caution should be applied to performing transposon mutagenesis screens without significant efforts exerted to validate findings. It also suggests that, despite the presence of a Tn*551* hotspot in the *S. aureus* genome, mutations identified within are no more, or less, likely to be artifacts for the phenotypes assessed.

## Conclusions

Herein, we present evidence that *sigS* expression is controlled by a number of factors within the *S. aureus* cell that are involved in amino acid biosynthesis, DNA-damage repair and cell wall structural integrity. In addition, we have identified a number of transcriptional regulators that influence *sigS* expression, at least two of which (CymR and KdpE) do so by direct binding to the promoter of this gene. The findings of the present study correlate with our previous works on σ^S^, which show a role for this regulator in the DNA-damage response, and in the resistance to cell wall targeting antibiotics and amino acid starvation [7,8]. Taken together, these results confirm our contention that *sigS* expression is tightly regulated within the *S. aureus* cell in a complex and multifactorial manner. A continued exploration of the role of this enigmatic protein remains a primary focus of our laboratory’s research endeavors.

## Additional file

Additional file 1: Table S1 Primers Used to Confirm NTML Strains. **Table S2.** Proteins identified as binding to the *sigS* promoter region in a biotin pull down assay. **Table S3.** Tn*551* Insertions Resulting in Increased Expression of *sigS.* **Table S4.** Tn*551* Insertions Resulting in Decreased Expression of *sigS.*

## Abbreviations

X-GAL: 5-bromo-4-chloro-2-indolyl-β-D-galactopyranoside
EMSA: Electrophoretic mobility shift assay
ECF: Extracytoplasmic function
h: Hour
ISPs: Ion Sphere Particles
IPTG: Isopropyl-1-thio-β-D-galactopyranoside
LB: Luria-Bertani
MMS: Methyl methanesulfonate
NTML: Nebraska Transposon Mutant Library
NARSA: Network on Antimicrobial Resistant in *Staphylococcus aureus*
Ni-NTA: Nickel-nitrilotriacetic acid
MNNG: N-methyl-N'-nitro-N-nitrosoguanidine
PGM: Personal Genome Machine
ROS: Reactive oxygen species
RNAP: RNA polymerase
SNPs: Single nucleotide polymorphisms
TSB: Tryptic soy broth
