# Supplementary material for: Investigating the genetic regulation of the ECF sigma factor σS in Staphylococcus aureus
Source: BMC Microbiol. 2014 Nov 30;14:280. doi: 10.1186/s12866-014-0280-9 (PMC4265319; doi:10.1186/s12866-014-0280-9)
Supplement: Additional file 1: Table S1 — Primers Used to Confirm NTML Strains. Table S2. Proteins identified as binding to the sigS promoter region in a biotin pull down assay. Table S3. Tn551 Insertions Resulting in Increased Expression of sigS. Table S4. Tn551 Insertions Resulting in Decreased Expression of sigS. [file 12866_2014_280_MOESM1_ESM.pdf]

Supplemental Table S1. Primers Used to Confirm NTML Strains.

Primer	Description	Gene
OL338	ATG GGA TCC GTT TCT GCT ATG TAC AAC	Forward *brnQ3*
OL339	ATG GAA TTC CCT AGG AAA GCG AAG CCA TTC	Reverse *brnQ3*
OL1573	TAG CGC TGA CGA ATG AAA CTT	Forward *sbcC*
OL1574	CAT CGC GTT TGC TTT ATT CAT C	Reverse *sbcC*
OL1633	GAA GGT CAA GCT GTT GAG	Forward *msa*
OL1634	AAG CAT CAT ATA CAT ACC ACA CT	Reverse *msa*
OL1635	CCG AAG TGC GAT GTT CAC TT	Forward *msrA*
OL1636	TCC AAG TGC TAA GAG GTA TAC AG	Reverse *msrA*
OL1652	GAA GTT GTG CAA GTG ATA CAT G	Forward *sacol1602*
OL1653	CGA CCG CTC TGT GCG ACT TGC TG	Reverse *sacol1602*
OL1654	GAG ACG GGA GAT TTC TAC GAG CC	Forward *glpF*
OL1655	GAG CTT GTT GTT CCT TGG TC	Reverse *glpF*
OL1656	CAA CAA GAT AAG GAG TGA AC	Forward *ebh*
OL1657	GTG ATG TCA ATC GGT GCT CCT C	Reverse *ebh*
OL2074	CAA GGT GCA TGG TTA TAT GTC	Forward *sucB*
OL2075	CAG CAG TAA GAT ATT TCG ACT AGG	Reverse *sucB*
OL2089	GAA CCA GGG TGA TGC AAT TCG	Forward *ctpA*
OL2090	GGA GGC GTG TCA TTA AC	Reverse *ctpA*
OL2469	GTC GGT GAA GTA CTT GGA ACT GG	Forward *hexA*
OL2470	CTG TAG CGC CTG CAT CTA TAG C	Reverse *hexA*
OL2471	GGT GTA TCA CCA GCA AAT GCT CC	Forward *tenA*
OL2472	GAT CAG TAC CTG CAA TGG TTA ATG C	Reverse *tenA*
OL2473	CTG TTT ACG CAG CAT ATA CCG CAC	Forward *femB*
OL2474	GCC TAT AGT ATC AAT AAC TAT AAG CC	Reverse *femB*
OL2475	GTC AAC TAT CCT AAG CGG AG	Forward *pbp2*
OL2476	CTG AGG ATT TTA TGT TGA GTG G	Reverse *pbp2*
OL2477	CAT TGC GGG TCA AGA TGT ATC AG	Forward *argJ*
OL2478	GAA CGA TAA TGG GGT AGA TGT TG	Reverse *argJ*
OL2479	GTA CAC ATT AGC TGT GAC TAA TG	Forward *sucA*
OL2480	GGA ACT TTA ACC TCT GGC ATG AC	Reverse *sucA*
OL2551	GTC AAG GCG TAG ATA TCC TAT GTA AGG	Forward *nupC*
OL2552	CAG ACA TAT TGT GCA TGT ATA TCA CC	Reverse *nupC*
OL2553	TGG TGG TGG GTT TAT TGG	Forward *sacol0682*
OL2554	CGT GCT AAT GCT AGA TGT	Reverse *sacol0682*
OL2555	AGA TAA AGA TGG GGA GGA	Forward *sacol0684*
OL2556	TAC ACT TAA CAC ACC CAC	Reverse *sacol0684*
OL2557	GGT TGG AAA GGT TGG GTT	Forward *sacol0940*
OL2558	GTA GTT GGT GAT TGT GCT G	Reverse *sacol0940*
OL2559	CCT CAT CTA AAC CTA TCG	Forward *sacol1349*
OL2560	TCC CCT CTT TTC TCT ATT TC	Reverse *sacol1349*
OL2561	TTT GCC CGT TTC TCT ACT	Forward *rpmG*
OL2562	CTT CGT AAT CAC CTT TTG CT	Reverse *rmpG*
OL2563	CGG TGT TGA AGA TAT TGG T	Forward *sacol1392*
OL2564	GCC ATT CCC TCT TTT TTG T	Reverse *sacol1392*
OL2565	AGA AGA TGG TAA GAA GGG	Forward *licT*
OL2566	AAG AGA CGA TGA TAG GTG	Reverse *licT*
OL2567	GTG AGA ATG ATG GCT TTG	Forward *sacol1412*
OL2568	GGA TTA GAT GAA GAG GAA GA	Reverse *sacol1412*
OL2569	ACC CGG CAC AAA TTA CAA	Forward *opp2F*
OL2570	AAC GGG GTG AAT ATG ATG A	Reverse *opp2F*
OL2571	CAA CCG GAG AGC AAC AAA	Forward *asd*
OL2572	GCC AAC ACC CTC AAA TAA A	Reverse *asd*
OL2573	AAG GCA TCA CAT ATC CAG AA	Forward *alr2*
OL2574	TAC CAA AGC CAC CAC CAA	Reverse *alr2*
OL2575	CAA CTC TAA TAG CAA CAC CA	Forward *sacol1445*
OL2576	CAC ACT CAA TAG CAA CCA	Reverse *sacol1445*
OL2577	CCG CCA ACT AAT CCA GAA	Forward *sacol1447*
OL2578	CAC AAA CAA CAC AAC AAG CA	Reverse *sacol1447*
OL2579	ATA GAA CAT CCC CCC CCA	Forward *sacol1452*
OL2580	GAC CAA GAC CAA GCA GAA	Reverse *sacol1452*
OL2581	TTG TTG TTT CGC TTG TGG T	Forward *ald*
OL2582	GAT GGA TAT GCG GGT GTT	Reverse *ald*
OL2583	CAC ACC TTC TTC TTC ATA C	Forward *sacol1479*
OL2584	CCA AAT CCT ACT ACA GCA	Reverse *sacol1479*

OL2585	ATC CCA ACA TAC AGC TAC A	Forward sacol*1480*
OL2586	AAC GAA CCA AAG GGG AAA	Reverse *sacol1480*
OL2587	CCA CCA GAA ACG CCC ATA	Forward *pepT2*
OL2588	TGC CAA CAA CAC ACA AAG	Reverse *pepT2*
OL2589	AAC CGC CAC CAG AAA TAA	Forward *sacol1556*
OL2590	TGC CAA CAA CAC ACA AAG	Reverse *sacol1556*
OL2591	CCG CAA AAT CCC CAT CAA	Forward *sacol1620*
OL2592	CCA TAA CTC ACC CCA ACC	Reverse *sacol1620*
OL2593	ACC CCT CCT AAA AAC CAA	Forward *ribD*
OL2594	CAA ATC CAA TGC CTC CAA	Reverse *ribD*
OL2735	GAA AAA TAG CTA AGG GGA GT	Forward *sacol0722*
OL2736	TGC TTT GTC TTG GTC ATT C	Reverse *sacol0722*
OL2737	ACC TCA TCC GCC AAT TAA A	Forward *isdB*
OL2738	CAT CGT CAC ACT CAT AAC T	Reverse *isdB*
OL2739	CAA CGG AGG GGT AAA TCA	Forward *pepA3*
OL2740	AAG ACA CCA CAT AAC GCA	Reverse *pepA3*
OL2741	CTA ATA GGT GAG GCG CTT	Forward *trpF*
OL2742	GTC TGC TTT TGC CTC TTT	Reverse *trpF*
OL2743	CAA TAG CAG TCA GTT TCA AG	Forward *pepF2*
OL2744	CAG CAA GAC ATT TAG AAC G	Reverse *pepF2*
OL2745	CAT AGA TGG TGT TGA AGA AG	Forward *pstC*
OL2746	GGG AGT AAT AGA AAG AGG T	Reverse *pstC*
OL2747	AAC CTC TTT CTA TTA CTC CC	Forward *pstS*
OL2748	CCC AGC CAT TTT TGT ATT C	Reverse *pstS*
OL2749	CCT GCG CCA AAA AAC ATA	Forward *norD*
OL2750	GGA AGA TGA ACG TGA ACA A	Reverse *norD*
OL2751	CCA TCT CTA TTT GCC TCC	Forward *sacol1476*
OL2752	AAG GGT GTG GTA GGT TAA A	Reverse *sacol1476*
OL2753	AAA CAT AGC TCA TCA CCC	Forward *sacol1531*
OL2754	CAA TCT GCC TCT CTA ATA C	Reverse *sacol1531*
OL2755	GGC CCC GGA CTA TAA TTG A	Forward *sacol1947*
OL2756	GGC CCC AAC AAA GAG AAA	Reverse *sacol1947*
OL2757	CCG AGA ATC TTT AAC ACC A	Forward *ilvB*
OL2758	ACA AGG TCG TAG CAT TCA	Reverse *ilvB*
OL2759	ATT GAT GTT CGT CCG GTT	Forward *hmrA*
OL2760	GAG TGT AGG GTA AAA GGG AG	Reverse *hmrA*
OL2761	GGG GAA ATA TGT AAA CGA AC	Forward *sacol2143*
OL2762	GGC CAA AGT ATT CAA CAA G	Reverse *sacol2143*
OL2763	AAG ACC GTG ATG AGA AGA	Forward *rocF*
OL2764	ACA TAG AGC AAA GGG GGA	Reverse *rocF*

Supplemental Table S2. Proteins identified as binding to the *sigS* promoter region in a biotin pull down assay.

Accession #	Gene	Function
**SACOL0009**	*serS*	Seryl-tRNA synthetase
**SACOL0435**	*ychF*	GTP-binding protein
**SACOL0476**	*hsdM*	Type I restriction-modification system M subunit
**SACOL0477**		Putative restriction/modification system specificity protein
**SACOL0562**	*lysS*	Lysyl-tRNA synthetase
**SACOL0570**	*clpC*	ATP-dependent Clp Protease ATP-binding subunit
**SACOL0588**	*rpoB*	DNA-directed RNA polymerase subunit beta
**SACOL0589**	*rpoC*	DNA-directed RNA polymerase subunit beta’
**SACOL0793**		Ribonucleoside-diphosphate reductase, beta subunit
**SACOL1215**	*carB*	Carbamoyl-phosphate synthase large chain
**SACOL1267**	*topA*	DNA topoisomerase 1
**SACOL1271**	*hslU*	ATP-dependent protease ATPase subunit
**SACOL1390**	*parC*	DNA topoisomerase IV, subunit A
**SACOL1494**	*asnS*	Asparaginyl-tRNA synthetase
**SACOL1614**	*nfo*	Probable endonuclease 4
**SACOL1681**	*cymR*	Rrf2 family protein
**SACOL1729**	*thrS*	Threonyl-tRNA synthetase
**SACOL1737**	*polA*	DNA polymerase I
**SACOL1759**		Putative universal stress protein
**SACOL1790**	*murC*	UDP-N-acetylmuramate-L-alanine ligase
**SACOL2213**	*rpoA*	DNA-directed RNA polymerase subunit alpha

Supplemental Table S3. Tn*551* Insertions Resulting in Increased Expression of *sigS*.

Accession Number	NE Number[a]	Tn*551* Insertion Site	Gene	Hits[b]	Unique[c]
		**DNA metabolism: DNA replication, recombination and repair**			
SACOL0566	NE544	Nucleoside permease	*nupC*	1	1
SACOL1315	NE974	DNA mismatch repair protein	*hexA*	1	1
SACOL1382	NE1451	Exonuclease	*sbcDC*	3	2
SACOL1413	N/A	Hypothetical protein (putative helicase		1	1
SACOL1479	NE789	Putative, 5'-3' exonuclease		2	1
		**Regulators**			
SACOL0513	NE1883	Transcriptional regulatory protein	*glcT*	1	1
SACOL1136	N/A	Hypothetical Protein (nucleic acid binding domain)		1	1
SACOL1393	NE1415	Transcriptional antiterminator LicT, putative	*licT*	3	1
SACOL1451	NE1684	Response regulator	*arlR*	1	1
SACOL1436	NE9	Modulator of SarA	*Msa*	1	1
SACOL2086	NE1218	Transcriptional regulator, TenA family	*tenA*	1	1
SACOL2188	NE436	Lactose phosphotransferase system repressor	*lacR*	5	1
		**Toxin production and resistance**			
SACOL1441	N/A	Tellurite resistance protein, putative		3	1
		**Cell envelope associated**			
SACOL1411	N/A	Aminoacyltransferase	*femB*	1	1
SACOL1472	NE1	Cell wall associated fibronectin-binding protein	*ebh*	6	2
		**Transporters**			
SACOL0682	NE1139	Putative antiporter		1	1
SACOL0684	NE1292	Na+/H+ antiporter, MnhE component, putative		2	1
SACOL0993	NE1633	Oligopeptide ABC transporter, ATP-binding protein	*oppD2*	1	1
SACOL1319	NE1580	Glycerol uptake facilitator protein	*glpF*	5	1
SACOL1392	NE142	Sodium:alanine symporter family protein		3	1
SACOL1414	NE1609	Peptide ABC transporter, ATP-binding protein	*opp2F*	4	1
SACOL1416	N/A	Peptide ABC-transporter, permease protein, putative		1	1
SAACOL1443	NE44	Branched-chain amino acid transport system II carrier protein	*brnQ3*	3	1
		**Amino acid biosynthesis**			
SACOL1680	NE595	Glutamate N-acetyltransferase/amino-acid acetyltransferase	*argJ*	2	1
SACOL1439	NE809	Hypothetical protein (Aspartate aminotransferase superfamily)		1	1
SACOL1429	NE1144	Aspartate-semialdehyde dehydrogenase	*asd*	1	1
SACOL1434	NE799	Alanine racemase family protein/diaminopimelate decarboxylase	*alr2*	1	1
SACOL1448	NE1391	Dihydroipoamide succinyltransferase	*sucB*	1	1
SACOL1449	NE547	2-Oxoglutarate dehydrogenase, E1 component	*sucA*	3	1
SACOL1478	NE1136	Alanine dehydrogenase	*ald*	1	1
SACOL2045	NE1177	Ketol-acid reductoisomerase		1	1
		**Protein synthesis and modification**			
SACOL1369	NE1904	50S ribosomal protein l33	*rpmG*	4	1
SACOL1397	NE1813	Peptide methionine sulfoxide reductase	*msrA*	4	1
SACOL1455	NE847	Carboxyl-terminal protease	*ctpA*	1	1
SACOL1480	NE582	Hypothetical protein (ribosome associated GTPase domain)		1	1
		**Unknown function**			
SACOL1452	NE577	PAP2 family protein		1	1
SACOL1602	NE377	Metallo-β-lactamase family protein		1	1
		**Hypothetical proteins**			
SACOL0940	NE1505	Hypothetical protein (No conserved domains)		1	1
SACOL1469	N/A	Hypothetical protein (No conserved domains)		1	1
SACOL1556	NE1346	Hypothetical protein (No conserved domains)		1	1
SACOL2642	N/A	Hypothetical protein (No conserved domains)		1	1
		**INTERGENIC**			
SACOLL1369/ SACOL1370	N/A	50S ribosomal protein L33/30S ribosomal protein S14	*rpmG/*	1	1
SACOL1396/ SACOL1397	N/A	fmtC protein/peptide methionine sulfoxide reductase	*fmtC/ msrA*	1	1
SACOL1412/ SACOL1413	N/A	Hydrolase-related protein/hypothetical protein (putative helicase)		2	1
SACOL1442/ SACOL1443	N/A	IS1272-related, transposase, degenerate/Branched-chain amino acid transport system II carrier protein	*/brnQ3*	2	1
SACOL1445/	N/A	CbbQ/NirQ/NorQ/GpvN family protein/ Conserved		1	1

SACOL1446		hypoethical protein			
SACOL1447/ SACOL1448	N/A	Conserved hypothetical protein/dihydroipoamide accinyltransferase		1	1
SACOL1449/ SACOL1450	N/A	2-oxoglutarate dehydrogenase, E1 component/Sensor histidine kinase	*sucA/ arlS*	2	1
SACOL1451/ SACOL1452	N/A	DNA-binding response regulator ArlR/PAP2 family protein	*arlR/*	1	1
SACOL1555/ SACOL1556	N/A	Peptidase, M20/M25/M40 family/hypothetical protein	*pepT2/*	1	1
SACOL1620/ SACOL1621	N/A	Conserved hypothetical protein/CBS domain protein		1	1
SACOL1820/ SACOL1821	N/A	Riboflavin biosynthesis protein RibD/Conserved hypothetical protein	*ribD/*	1	1
SACOL1893/ SACOL1894	N/A	ABC transporter, ATP-binding protein/HIT family protein		1	1

[a]NE numbers are the identification number in the Nebraska Transposon Mutant Library.
[b]Hits refer to the total number of Tn*551* insertion sites identified for a particular gene.
[c]The unique number of insertions sites refers to those insertions that are a result of distinctive insertion events.
N/A means there was no NTML strain for this gene

Supplemental Table S4. Tn*551* Insertions Resulting in Decreased Expression of *sigS*.

Accession Number	NE Number[a]	Tn551 Insertion Site	Gene	Hits[b]	Unique[c]
		**Regulators**			
SACOL1393	NE1415	Transcriptional antiterminator, *licT* putative	*licT*	3	1
SACOL1398	N/A	Transcriptional regulator, putative		1	1
		**Cell Envelope Associated**			
SACOL1138	NE1102	LPXTG cell wall surface anchor protein	*isdB*	1	1
SACOL1531	NE707	Lipoprotein, putative		1	1
		**Transporters**			
SACOL0722	NE187	Phosphate transporter family protein		1	1
SACOL1424	NE1459	Phosphate ABC transporter, phosphate binding protein	*pstS*	2	1
SACOL1443	NE44	Branched chain amino acid transport system II carrier protein	*brnQ3*	7	1
SACOL1476	NE211	Amino acid permease		1	1
		**Amino Acid Biosynthesis**			
SACOL1407	NE1215	5'Phosphoribosyl anthranilate isomerase	*trpF*	1	1
SACOL2043	NE1166	Acetolactate synthase, large subunit	*ilvB*	1	1
SACOL2154	NE134	Arginase	*rocF*	1	1
		**Protein Synthesis and Modification**			
SACOL1402	NE1306	Glutamyl aminopeptidase, putative	*pepA3*	1	1
SACOL1419	NE195	Oligoendopeptidase F, putative	*pepF2*	1	1
SACOL2125	NE600	Peptidase, M20/M25/M40 family protein	*hmrA*	1	1
		**Unknown Function**			
SACOL1412	NE1611	Hydrolase-related protein		2	1
SACOL1452	NE577	PAP2 family protein		1	1
		**Hypothetical Proteins**			
SACOL1344	N/A	Hypothetical protein		3	2
SACOL1418	N/A	Conserved hypothetical protein		1	1
SACOL1444	NE143	Conserved hypothetical protein		3	2
SACOL1947	NE1706	Conserved hypothetical protein		3	1
SACOL2143	NE1104	Conserved hypothetical protein		2	1
		**INTERGENIC**			
SACOL0610/ SACOL0611	N/A	SdrE protein/glycosyl transferase, group 1 family protein	*sdrE/*	1	1
SACOL1348/ SACOL1349	N/A	Conserved hypothetical protein/conserved hypothetical protein		1	1
SACOL1369/ SACOL1370	N/A	Ribosomal protein L33/ribosomal protein S14	*rpmG1 /rpsN1*	1	1
SACOL1398/ SACOL1399	N/A	Transcriptional regulator, putative/ 4-oxalocrotonate tautomerase	*/dmpI*	1	1
SACOL1407/ SACOL1408	N/A	5'Phosphoribosyl anthranilate isomerase/tryptophan synthase, beta subunit	*trpF/tr pB*	1	1
SACOL1411/ SACOL1412		FemB protein/hydrolase related protein	*femB/*	1	1
SACOL1441/ SACOL1442	N/A	Tellurite resistance protein, putative/ IS1272-related transposase		1	1
SACOL1443/ SACOL1444	N/A	Branched-chain amino acid transport system II protein/Conserved hypothetical protein	*brnQ3/*	1	1
SACOL1447/ SACOL1448	N/A	Conserved hypothetical protein/2-oxoglutarate dehydrogenase, E2 component	*/sucB*	1	1
SACOL1468/ SACOL1469	N/A	Membrane protein, putative/hypothetical protein		1	1
SACOL1711/ SACOL1712	N/A	DNA 3-methyladenine glycosylase /arbB protein putative		1	1
SACOL1913/ SACOL1914	N/A	RNA methyltransferase/Iron-sulfer cluster, binding protein		1	1

[a]NE numbers are the identification number in the Nebraska Transposon Mutant Library.

[b]Hits refer to the total number of Tn*551* insertion sites identified for a particular gene.
[c]The unique number of insertions sites refers to those insertions that are a result of distinctive insertion events.
N/A means there was no NTML strain for this gene
